# Incidence of COVID-19 infection in hospital workers from March 1, 2020 to May 31, 2021 routinely tested, before and after vaccination with BNT162B2

**DOI:** 10.1038/s41598-021-04665-y

**Published:** 2022-02-15

**Authors:** Francesca Larese Filon, Francesca Rui, Federico Ronchese, Paola De Michieli, Corrado Negro

**Affiliations:** Unit of Occupational Medicine, University of Trieste, Via della Pietà 2/2, 34129 Trieste, Italy

**Keywords:** Diseases, Health occupations

## Abstract

To evaluate the incidence of COVID-19 infection in health care workers from the start of the COVID-19 pandemic in NE Italy, vaccination with BNT162b2. This was a retrospective cohort study. Healthcare workers were routinely tested for SARS-CoV-2 infection using real-time polymerase chain reaction tests in nasopharyngeal swabs. Logistic regression was used to calculate the incidence rate ratios (IRRs) of the factors associated with COVID-19. A total of 4251 workers were followed up, and the prevalence of COVID-19 was 13.6%. In March 2021 the incidence of infection was 4.88 and 103.55 cases for 100,000 person-days in vaccinated and non-vaccinated workers, respectively, with an adjusted IRRs of 0.05 (95% CI 0.02–0.08). Our study evaluated the monthly incidence in health care workers in Trieste hospitals before and after vaccination, finding an estimated vaccine effectiveness of 95% in health care workers routinely tested.

## Introduction

The first index case was identified on March 1, 2020, at Trieste Hospital (Italy) in the geriatric ward. Since then, hospital workers were screened for SARS-Cov-2 infection in the first month after contact with cases or in case of symptoms and then^1^, on a weekly or monthly basis, for workers in COVID-19 or non-COVID-19 wards, respectively. On December 27, 2020, a large campaign was launched in Italy to vaccinate its population, and health care workers (HCWs) were among the first eligible for vaccination, along with individuals at risk of COVID-19 complications and death^2^. In Friuli Venezia Region, hospital workers started to be vaccinated at the same time as the launch, and by the end of February, 2021 72% of HCWs in Trieste hospitals received two doses of vaccination if never infected by SARS-CoV-2 and one dose in case of previous infection 3–6 month before.

The BNT162b2 COVID-19 vaccine manufactured by Pfizer and BioNtech demonstrated 95% efficacy in preventing symptomatic SARS-CoV-2 infection in a phase 3, placebo-controlled randomized clinical trial^3^, which is the first COVID-19 vaccine to receive emergency use authorization by the US Food and Drug Administration^4^ and European Medicines Agency^5^. The risk ratio for symptomatic disease, estimated from a cohort of more than 500,000 vaccinated adults and matched unvaccinated controls was 0.06^6^. However, the association between the BNT162b COVID-19 vaccine and asymptomatic infection is crucial, and data are scanty^7^.

Data on the incidence of COVID-19 infection from the start of the pandemic before and after vaccination are limited^7,8^, and none are available for Italy. Previous authors^7,8^ reported a significantly lower incidence of symptomatic and asymptomatic SARS-CoV-2 infection after vaccination with an incidence rate ratio adjusted of 0.03 (95% CI 0.01–0.06) and 0.14 (95% CI 0.07–0.31), respectively.

The aims of our study were to evaluate:

1. The incidence of COVID-19 infection in hospital workers in Trieste (NE, Italy) before the vaccination from March 1, 2020, to February 28, 2021 (phase I of the study).
2. The incidence of COVID-19 after vaccination from March 1, 2021, to May 31, 2021 (phase II of the study).

## Methods

This was a retrospective cohort study designed to estimate the incidence of COVID-19 infection among hospital workers in Trieste public hospitals. The study was conducted in Azienda Sanitaria Universitaria Giuliana Isontina (ASUGI) in Maggiore and Cattinara hospitals, a tertiary medical center that employs approximately 4300 workers. Since April 15, 2020, hospitals have implemented a policy of routine screening for all workers using nasopharyngeal swabs and PCR-based virus detection. In addition, each case of SARS-CoV-2 infection in health care workers (HCWs) or patients triggered an epidemiological investigation that included the repetition of nasopharyngeal swabs and the collection and recording of symptoms. All hospital workers can receive vaccination from the first day of the vaccination campaign and throughout the study period on a priority basis.

Data were retrieved from the hospital information system database, which aggregates data from multiple sources, including personnel files, vaccination reports, laboratory databases, and digitized epidemiological questionnaires.

Ethics approval and review were performed according to the Declaration of Helsinki and were obtained from the Comitato Etico Unico Regionale (CEUR) along with a waiver of written informed consent.

### Definition of follow-up period for the incidence of COVID-19 in hospital workers (phase 1 of the study)

The follow-up started with the first index case identified in Trieste hospitals on March 1, 2020, and continued until May 31, 2021.

PCR tests were performed according to the screening policies of the hospitals. The screening policy changed during the course of the study as follows: in March, only health care workers (HCWs) with respiratory symptoms or in contact with a COVID-cases were tested (period 1); from April 15, 2020, to May 31, 2021, all hospital workers, including technicians and administrative staff, were screened depending on their risk of SARS-CoV-2 exposure (weekly for HCWs in COVID-19 wards and monthly for the others). After the start (December 27, 2021) routine screening continued with the same protocol.

All hospital workers had open and free access to PCR testing at their own discretion. Hospitals workers who did not undergo at least one PCR test during the study period were excluded from the analysis. Workers who contracted SARS-CoV-2 infection during the study contributed to the group of COVID-19 workers, while the others were assigned to the COVID-19 negative (−) group.

To calculate the incidence of COVID-19, workers with previous SARS-CoV-2 infection was excluded from the analysis.

### Group assignment for evaluate the effectiveness of vaccination (phase II of the study)

Hospitals workers who received at least one vaccine dose between December 27, 2020, and February 28, 2021, were assigned to the vaccinated group. The control group consisted of hospital workers who did not receive any doses of the BNT162b2 vaccine during this period.

To calculate the incidence of COVID-19 in vaccinated and non-vaccinated workers, subjects with previous COVID-19 infection were excluded from the analysis.

The follow-up period for each worker was defined as starting from March 1, 2021, to May 31, 2021. Participants were censored at the first positive PCR test results, or in May, 31.

### Definitions

The risk of exposure to COVID-19 subjects was classified as high for persons working in COVID-19 dedicated wards, medium for persons working in Internal Medicine I and II (where inpatients with respiratory symptoms were hospitalized waiting for a diagnosis), low for workers in other wards, and very low for administrative staff and technicians. In case of exposure to various risk situations, the highest risk level of exposure was used.

Participants with COVID-19 were defined as symptomatic if they had any of the following: temperature greater than 37.5 °C, upper respiratory symptoms (sore throat, cough, and rhinorrhea), lower respiratory symptoms (pneumonia, dyspnea), loss of sense of taste or smell, diarrhea, others (myalgia, malaise, and headache). Symptoms were obtained during the first PCR test and from post-infection epidemiological interviews performed by the unit of occupational medicine involved in infection and prevention control. The participants were considered fully vaccinated for more than seven days after the second dose of the vaccine.

### Study outcomes

The primary outcome was the incidence of SARS-CoV-2 infection in hospital workers from the start of the pandemic to February 28, 2021. The secondary outcome was the incidence of SARS-CoV-2 infection from March 1 to May 31, 2021, in vaccinated and non-vaccinated workers. Outcomes were analyzed for pre-defined subgroups according to age, sex, work task, and wards according to the estimated level of exposure.

### Statistical analysis

Continuous variables are expressed as mean and standard deviation (SD) for normally distributed variables and as median (interquartile range) for non-normally distributed variables. Categorical variables were expressed as the number (percentage) of hospital workers within each group. The groups were compared using the t-test for normally distributed continuous variables and chi-square tests for categorical variables. Missing values for categorical variables were considered as separate categories. A significance for p < 0.05 two sided was considered significant.

### Incidence rate analysis

The incidence rates were defined as the number of positive SARS-CoV-2 cases divided by the number of person—days during the surveillance period.

The adjusted incidence rate ratio (IRR) was estimated using a multivariate Poisson regression model with confirmed cases as a response variable and group assignment (COVID-19 positive and negative for study phase I and vaccinated and non-vaccinated for study phase II), age sex work tasks, wards, and number of PCR tests for each health care worker in the time frame under observation. The IRRs were computed by exponentiating the group assignment coefficient from the final regression model, and the 95% CI s and P values were estimated from the model.

All statistical analyses were performed using STATA (StataCorp LLC, College Station, TX, USA).

## Results

### Phase I study

#### Incidence of COVID-19 acquired infection from March 1, 2020 to February 28, 2021

**Figure 1:**
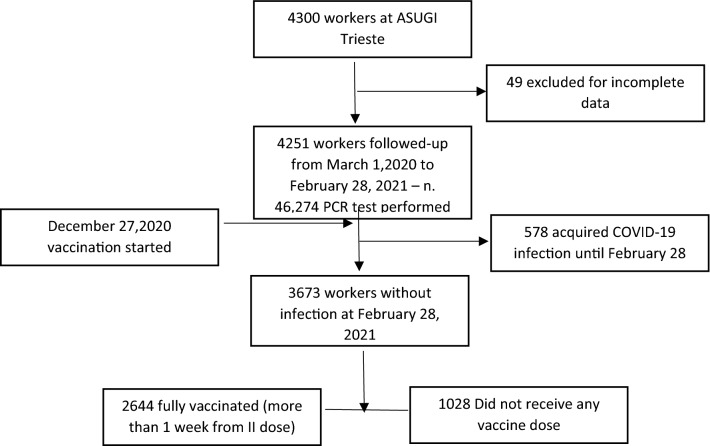
Study cohort analyzed (PCR = polymerase chain reaction in nasopharyngeal swabs).

**Figure 2:**
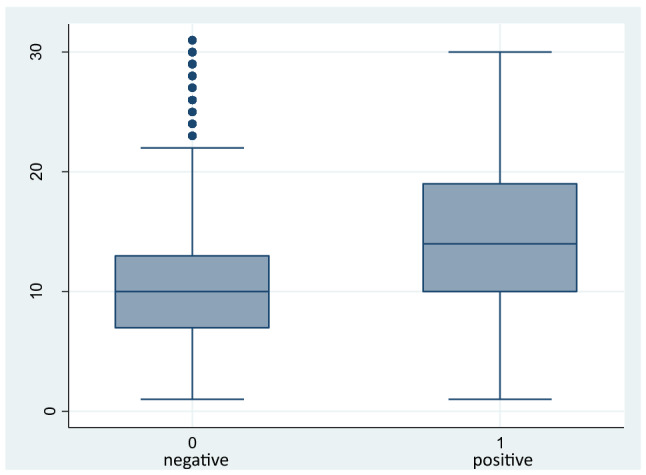
Box-plot analysis of numbers of swabs performed in negative (0) and positive (1) workers. Data are reported as median, 25°–75° percentile, minimum and maximum and outliers.

A 1-year incidence study was performed on a cohort of HCWs at Trieste Hospitals (Maggiore and Cattinara) comprising 4300 workers (Fig. 1). Forty-nine workers were excluded for incomplete data, while 4231 workers were followed up from the start of the SARS-CoV-2 pandemic in Trieste (Italy) for 12 months. During this period, 46,274 PCR tests were performed in nasopharyngeal swabs of HCWs that were routinely screened to detect early COVID-19 infection. The vaccination campaign in Italy for HCWs started on December 27 and before February 20, 2021; 72% of the workforce received two vaccination doses. During the 12 months period 578 HCWs acquired COVID-19 (13.6%). Table 1 reports the characteristics of the cohort. The sex distribution was similar for infected and non-infected workers, but COVID-19 HCWs were significantly younger (44.7 ± 11.1 years vs 47.2 ± 10.6 years, p < 0.001, respectively), and subjects with less than 31 years of age had a greater effect (p < 0.001). The majority of workers who acquired the infection were nurses (45.2%) and nurse aids (21.8%). Prevalence of infection differed in relation to exposure risk: in some wards involved in the treatment of COVID-19 patients, infected workers were more than 50% (long-term Covid) or less beyond (geriatric and infectious diseases with 46.5% and 47.9% of the workforce that resulted in infection). In other divisions such as Rehabilitation and Pulmonology, both COVID-19 wards, 21.1% and 30.5% of HCWs acquired the infection. Lower percentages of COVID-19 were found in other wards not involved in the treatment of COVID-19 patients such as surgeries (10.5%) and other medicine wards (7.3%). Notably, HCWs in Anesthesia and Resuscitation, involved in the treatment of COVID-19 patients, presented a very low prevalence of SARS-CoV-2 infection (8.6%). In the overall cohort, each worker performed 10 PCR tests each as median (25–75° percentiles 7–14), with a higher number of tests for infected HCWs that repeated PCR more often (median 14 tests, 25–25° percentiles 10–19), p = 0.03. Box-plot data distribution of tests performed is reported in Fig. 2. Data are reported as median, 25°–75° percentile, minimum and maximum and outliers.

**Table 1:** Characteristics of the population studied and acquired COVID-19 infection from March 1, 2020 to February 28, 2021.

	Covid-19+	Covid 19−	Total	p
Total n. (%)	578 (13.6)	3673 (86.4)	4.251 (100)	
Women n. (%)	394 (68.2)	2550 (69.4)	2.944 (69.3)	0.542
Age mean (SD)	44.7 ± 11.1	47.2 ± 10.6	46.9 ± 10.7	0.000
**Age classes n. (%)**				
< 31	87 (15.1)	335 (9.1)	422 (9.9)	0.000
31–40	115 (19.9)	660 (18.0)	775 (18.2)	
41–50	170 (29.4)	1061 (28.9)	1231 (29.0)	
51–60	175 (30.3)	1325 (36.1)	1500 (35.3)	
> 60	31 (5.4)	292 (7.9)	323 (7.6)	
HCWs n. (%)	522 (90.3)	3232 (88.0)	3755 (88.3)	0.110
Non-HCWs n. (%)	56 (9.7)	440 (12.0)	496 (11.7)	
Physicians n. (%)	78 (13.5)	539 (14.7)	617 (14.5)	0.000
Nurses n. (%)	261 (45.2)	1459 (39.7)	1720 (40.5)	
Nurses aids n. (%)	126 (21.8)	619 (16.9)	745 (17.5)	
Others HCW n. (%)	57 (9.9)	615 (16.7)	673 (15.8)	
Technicians n. (%)	20 (3.5)	201 (5.5)	221 (5.2)	
Clerks n. (%)	36 (6.2)	239 (6.5)	275 (6.5)	
**Wards**				
Geriatric I n. (%)	20 (46.51)	23 (53.49)	43 (100)	0.000
Infective diseases n. (%)	23 (47.9)	25 (52.1)	48 (100)	
Long-term Covid n. (%)	26 (57.8)	19 (42.2)	45 (100)	
Medicine I and II n. (%)	57 (27.9)	147 (72.1)	204 (100)	
Emergency medicine n. (%)	18 (30.5)	41 (69.5)	59 (100)	
Pulmonology n. (%)	26 (21.85)	93 (78.15)	119 (100)	
First aids n. (%)	51 (18.75)	221 (81.25)	272 (100)	
Rehabilitation n. (%)	22 (22.2)	77 (77.8)	99 (100)	
Others n. (%)	126 (9.7)	1175 (90.3)	1301 (100)	
Surgeries n. (%)	95 (10.5)	814 (89.5)	909 (100)	
Administration/technicians n. (%)	50 (11.9)	371 (88.1)	421 (100)	
Anesthesia and resuscitation n. (%)	17 (8.6)	181 (91.4)	198 (100)	
Other medicines n. (%)	26 (7.3)	329 (92.7)	355 (100)	
Radiology and nuclear medicine n. (%)	21 (11.8)	157 (88.2)	178 (100)	
N° PCR tests per person, median (IQR)	14 (10–19)	10 (7–10)	10 (7–14)	0.03
Symptoms at the first PCR + n. (%)	301 (52.1)	–	301 (52.1)	
Symptoms recorded at the last PCR + n. (%)	483 (83.6)	–	483 (83.6)	
Asymptomatic n. (%)	95 (16.4)	–	95 (16.4)	
Upper respiratory symptoms n. (%)	315 (54.5)	–	315 (54.5)	
Fever > 37.5° C n. (%)	273 (47.2)	–	273 (47.2)	
Loss of taste and smell n. (%)	161 (27.8)	–	161 (27.8)	
Lower respiratory symptoms n. (%)	33 ( 5.7)	–	33 (5.7)	
Diarrhea n. (%)	35 ( 6.0)	–	35 (6.0)	
Others (myalgia, malaise, headache) (%)	249 ( 43)	–	249 (43)

**Figure 3:**
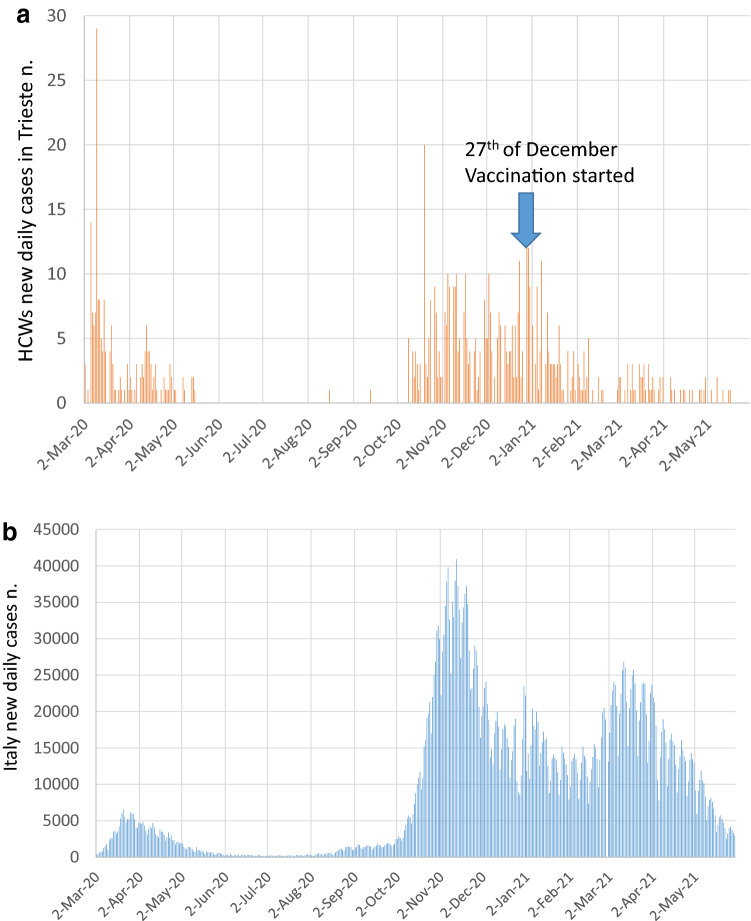
(**a**) Incidence cases of COVID-19 in Italian population from March 1, 2020 to May 31, 2021. (**b**) Incidence cases in health care workers (HCWs) from March 1, 2020 to May 31, 2021.

Figure 3a shows the daily cases of HCWs with SARS-CoV-2 infection from March 1, 2020, to May31, 2021 and Fig. 3b reports daily cases in Italy showing the two waves of infection that characterized our Country, with the first wave from March to May 2020 and the second wave from October 2020 to February 2021. The start of vaccination on December 27, 2020, caused a deep decline in cases until 0 in mid-February 2021. A total of 52.1% of subjects reported symptoms on the day of the first positive PCR, while the others were asymptomatic. During the follow-up, 16.4% of workers remained asymptomatic, while the others developed symptoms, mainly mild in the upper respiratory airways (sore throat, cough, rhinorrhea: 54.5%), fever > 37.5 °C (47.2%), and loss of taste and smell (27.8%). Thirty-three workers had lower respiratory symptoms (5.7%), and one patient died.

The monthly incidence of SARS-CoV-2 infection is reported in Table 2 as person-days, in March, 2020 the incidence was 72.69 cases per 100,000 person-days declining in April and May 2020. No cases were recorded in June and July, 2020 while few cases occurred in August and September 2020. The second wave of infection started in October 2020 with an incidence of 55.7 cases per 100,000 person-days, which increased to 97.57 and 93.97 cases per 100,000 person-days in November and December 2020, respectively. In January, the incidence was still high (61.12 cases per 100,000 person-days) and finally declined in February 2021 (13.56 cases per 100,000 person-days).

**Table 2:** Incidence of COVID-19 infection in hospital workers in Trieste from March 1, 2020 to February 28, 2021.

Month	Surveillance time, person-days	COVID-19 cases	Incident rate per 100,000 person-days	95 CI
March	130,696	95	72.69	64–79
April	123,870	71	57.32	48–66
May	126,077	15	11.90	6.8–19
June	121,680	0	0.00	–
July	125,705	0	0.00	–
August	125,705	4	3.18	0.9–7.9
September	121,650	8	6.58	3–12
October	125,674	70	55.70	46–64
November	119,910	117	97.57	93–99
December	120,249	113	93.97	88–98
January	116,157	71	61.12	52–70
February	103,236	14	13.56	8–22

Table 3 reports the incidence risk ratio (IRR) and 95% confidence intervals (CI) of factors associated with SARS-CoV-2 infection in HCWs calculated using Poisson regression analysis. Younger age was confirmed as a factor involved in COVID-19 acquired infection, together with work in some COVID-19 dedicated wards. The highest IRR was found for HCWs in the long-term cohort (IRR 7.15, 95% CI 4.6–11.1) compared to other medicine wards. Notably, anesthesia and resuscitation, a COVID-19 ward did not present an increased risk of infection.

**Table 3:** Incidence risk ratio (IRR) and 95 confidence intervals (CI) of factors associated with COVID-19 infection in hospitals’ workers calculated using the Poisson regression adjusted for age and number of PCR test performed.

Workers’ characteristics	Adjusted IRR (95% CI)	p
Age	0.99 (0.98–0.99)	0.012
**Sex**		
Men	0.94 (0.80–1.1)	0.422
Women	1	
**Work tasks**		
Physician	1	
Nurse	1.18 (0.94–1.49)	0.153
Nurse aid	1.18 (0.91–1.52)	0.203
Other HCW	0.70 (0.50–0.98)	0.036
Technician	0.72 (0.43–1.22)	0.223
Clerks	1.18 (0.79–1.76)	0.428
**Wards**		
Other medicines	1	
Anesthesia and resuscitation	1.10 (0.61–1.97)	0.751
Others	1.48 (0.98–2.23)	0.060
Surgeries	1.37 (0.90–2.08)	0.135
Radiology and nuclear medicine	1.96 (1.14–3.36)	0.015
Administration and technicians	1.85 (1.13–3.04)	0.014
First aid	2.44 (1.56–3.83)	0.000
Pneumology—Covid	2.72 (1.65–4.48)	0.000
Rehabilitation—Covid	3.59 (2.14–6.04)	0.000
Internal medicine and clinical medicine—pre-Covid	3.52 (2.28–5.42)	0.000
Emergency medicine	3.70 (2.15–6.37)	0.000
Geriatry—Covid	5.96 (3.64–9.76)	0.000
Infective diseases—Covid	5.75 (3.54–9.33)	0.000
Long term—Covid	7.15 (4.59–11.14)	0.000

### Phase II study

#### Incidence of COVID-19 acquired infection from March 1, 2021 to May 31, 2021

Since March 1, 2021, 72% of workers had been fully vaccinated against COVID-19 with the second shot done before the mid-February 20, 2021. The characteristics of the population are reported in Table 4. Women were less vaccinated than men (68% vs. 80.9%, p < 0.001) and non-HCWs resulted in less vaccination than HCWs (65.5% vs. 73.3%, p < 0.001). Physicians were significantly more vaccinated (87.0%) than the other professional groups.

**Table 4:** Characteristics of full vaccinated and non-vaccinated health care workers at March 1, 2021.

	Full vaccinated	Non-vaccinated	Total	p
Total n. (%)	3060 (72)	1191 (28)	4251 (100)	
Women n. (%)	2002 (65.4)	942 (79.1)	2944 (69.2)	0.01
Age mean (SD)	46.8 (11.3)	46.9 (11.2)	46.8 (11.2)	0.37
HCWs n. (%)	2732 (89.3)	1002 (84.1)	3755 (88.3)	0.001
Non-HCWs n. (%)	325 (10.6)	171 (14.3)	496 (11.7)	
Physicians n. (%)	537 (17.5)	80 (0.7)	617 (14.5)	0.001
Nurses n. (%)	1238 (40.4)	482 (40.4)	1720 (40.4)	
Nurses aids n. (%)	507 (16.6)	238 (20.0)	745 (17.5)	
Others HCW n. (%)	471 (15.4)	202 (17.0)	673 (15.8)	
Technicians n. (%)	144 (4.7)	77 (6.5)	221 (5.2)	
Clerks n. (%)	182 (5.9)	93 (7.8)	275 (6.5)	

In Table 5, the incidence of SARS-CoV-2 infection was compared between vaccinated and non-vaccinated workers. Only six vaccinated HCWs acquired the infection in the 3 months considered, while the incidence was very high in March for non-vaccinated people (103.55 cases per 100,000 person-days, declining in April (50.25 cases per 100,000 person-days) and May (13 cases per 100,000 person-days) in correspondence with the decline of the infection in the general population in Italy. The incidence rate ratio between vaccinated and non-vaccinated workers was 0.05 (95% CI 0.02–0.08) in March and April 2021.

**Table 5:** Incidence of COVID-19 infection (95% Confidence Intervals CI) in hospital workers in Trieste from March 1, 2021 to May 31, 2021 in vaccinated and non-vaccinated workers.

Month	Surveillance time, person-days	COVID-19 cases vaccinated		Surveillance time, person-days	COVID-19 cases non-vaccinated		Incidence rate ratio (95 CI)		p-value
	Vaccinated	n	Incident rate per 100,000 person-days	Non-vaccinated	n	Incident rate per 100,000 person-days	Unadjusted	Adjusted^a^	
March	81,964	4	4.88	31,868	33	103.55	0.05 (0.02–0.1)	0.05 (0.02–0.08)	< 0.001
April	79,200	2	2.53	29,850	15	50.25	0.05 (0.02–0.1)	0.05 (0.02–0.08)	< 0.001
May	81,778	2	2.45	30,721	4	13.02	0.19 (0.16–0.28)	0.20 (0.16–0.30)	< 0.01

^a^Using Poisson regression, as reported in the M&M section.

## Discussion

### Phase I study

Our study investigated the incidence of COVID-19 acquired infection in HCWs regularly screened for SARS-CoV-2 in nasopharyngeal swabs. The screening permitted to identify asymptomatic or pre-symptomatic infection, with the aim of reducing the transmission to colleagues and patients.

In France, the incidence rate was estimated to be 5%^9^ between March 17 and April 20, 2020, and in Italy, Colaneri et al.^10^ reported an incidence of 11.33% (9.72–13.21) from February 22 to May 8, 2020, and in Boston, Lan et al.^11^ reported a higher incidence (14%) between March 9 and April 15, but all authors tested only symptomatic workers. A literature review estimated a pooled prevalence of 11%, higher for symptomatic workers (19%), and lower for asymptomatic workers (5%)^12^.

Asymptomatically infected individuals are estimated to account for 40–45% of SARS-CoV-2 infections and may spread the virus over extended periods^13^. Asymptomatic infections have been proposed as a major barrier to controlling the spread of SARS-CoV-2 infection and are a possible explanation for the rapid evolution of the COVID-19 pandemic^13^.

In our cohort, 47.9% of subjects were asymptomatic on the day of the first PCR positive; during the follow-up of symptoms, 16.4% remained asymptomatic while the others developed symptoms, mainly on the upper airways. For this reason, the regular screening of workers using PCR SARS-CoV-2 detection in nasopharyngeal swabs was the first line (before vaccination) to control the spread of infection in hospital workers. However, analyzing numbers of swab tests performed, some workers underwent a lower number of tests than expected, this result could be explained by holiday periods, sick leave but also to a possible tendency to skip tests in some subjects.

In our study, the incidence of COVID-19 infection was 13.6% between March 1, 2020, and February 28, 2021, and Colaneri et al. reported a cumulative incidence of 11.33% in another Italian hospital analyzing infections from February 22 to May 8, 2020. The risk of being COVID-19 positive decreased with age (IRR 0.99; 95% CI 0.98–0.99) and people less than 31 years of age were more likely to be infected compared to older workers. Studies that tested only symptomatic workers did not find an increased risk in young people^9,10^, probably because they did not routinely test HCWs, losing younger subjects who had frequently asymptomatic infection. A similar risk of acquiring the infection was found in different job categories, with the exception of workers without direct contact with patients (“Others” IRR 0.70; 95% CI 0.50–0.98). Big differences were found in wards in which the highest risk was found in some COVID-19 dedicated wards (Long-term, Infective diseases, Geriatrics) and where occurred some infection clusters (Emergency medicine, Internal medicine), while the risk to acquire the infection was lower in “Other medicines,” in Surgeries, in Anesthesia and Resuscitation. This finding is similar to other reports in which COVID-wards presented a higher risk of infection for employed workers^9,10^. To evaluate the trend of infection during one year, we calculated the incidence per 100,000 person-days finding a wide variation, as happened in the regional general population in Italy and in our region; the pandemic started in March 2020 with 72.60 (95% CI 64–98) cases per 100,000 person-days and decreased to 0 in June and July, after the Italian lockdown. The second wave of the pandemic started slowly at the end of the summer and reached a peak in November and December with 97.57 (95% CI 93–99) and 93.97 (95% CI 88–98) cases per 100,000 person-days.

### Phase II study

Vaccination for all HCWs was available from December 27, 2020, and the decrease in COVID-19 infections started in February, with 0 cases from the second half of the month. The incidence in March, April and May 2021 was calculated considering vaccinated and non-vaccinated HCWs showing an extremely low number of cases in vaccinated people and the highest incidence in March, 2021 for non-vaccinated HCWs with an incidence of 103.55 cases per 100,000 person-days. The IRR between vaccinated and non-vaccinated was 0.05 (95% CI 0.01–0.08) in March and April 2021. Our results are similar to those reported by Angel in 2021^7^ in Israel, with an incidence rate of 4.5 for 100,000 person-days in fully vaccinated HCWs, while they found a higher incidence in non-vaccinated people (148.8 cases for 100,000 person-days). Tang et al.^8^ reported an incidence rate ratio for confirmed COVID-19 cases per person-days in vaccinated compared to unvaccinated group of 0.04 (95% CI 0.02–0.09).

Our study found an IRR of 0.05 for vaccinated compared to non-vaccinated HCWs, corresponding to an estimated vaccine effectiveness of (1-IRR) 95%, that is the same reported in the phase 3 randomized clinical trial^3^ and the risk ratio of 0.06 observed in a study of the nationwide vaccination in Israel^6^. Benenson et al.^14^ found a very low incidence of COVID-19 in vaccinated workers compared with non-vaccinated workers. The weekly incidence of Covid-19 since the first dose declined notably after the second week; the incidence of infection continued to decrease dramatically and then remained low after the fourth week. Amit et al.^15^ reported a reduction rate of 85% in vaccinated vs. non-vaccinated HCws.

Vaccination permitted reduction of COVID-19 infection in HCWs, and vaccine coverage of 72% of HCWs in our hospitals permitted the reduction of acquired infection in non-vaccinated HCWs in May 2021.

Moreover, the onset of new variants with higher infectivity and potentially lower effectiveness of vaccination is a crucial point to evaluate in the follow-up of our cohort, considering that from summer 2021, the majority of COVID-19 cases in Italy were related to variant Delta^18^.

### Effectiveness of the screening

Our study highlighted COVID-19 infection in HCWs in Italy from the start of the pandemic in our region (March 1, 2020) for one year and evaluated COVID-19 incidence of infection in fully vaccinated and non-vaccinated workers. To the best of our knowledge, our study is the first in Italy and one of the few in the world^16,17^ that evaluated the monthly incidence of COVID-19 infection from the start of the pandemic until now, using weekly or monthly PCR screening for all HCWs according to their risk of being exposed to COVID-19 infected persons. The screening permitted the identification of positive asymptomatic or pre-symptomatic subjects that need to be restricted from work to avoid the spread of infection to patients and colleagues.

The effectiveness of periodical screening is also demonstrated by the lower incidence of COVID-19 acquired infection in non-vaccinated people compared to other available data. Angel et al.^7^ reported an incidence in non-vaccinated HCWs of 149.8 cases for 100,000 person-day, which is higher than our data for March 2021 (103.55 cases for 100,000 person-day), although these authors also performed periodical screening in HCWs during the surveillance period.

The strength of our study is the long follow-up that permitted the definition of a monthly incidence of COVID-19 infection in HCWs before and after vaccination. Routine PCR detection in nasopharyngeal swabs permitted the identification of asymptomatic and pre-symptomatic infections to limit the spread of the infection to patients and other workers^19,20^.

### Limitations of the study

This study has several limitations. First, it was a single-center, retrospective cohort study, and our findings may not be generalizable. Second, the effect of vaccination was studied for three months in witch progressively decreased COVID-19 infection in our region. Third, other confounders may be present there were unaccounted for in the regression analysis, such as health status and relationship between health status and vaccination. Lastly, despite the high numbers of swab tests performed, it is possible that some workers skipped them during holidays or sick leave. In addition, we are confident that our workers did not performed additional tests outside the hospital because tests were very expensive that time.

## Conclusions

Our study reported data on the incidence of COVID-19 infection in a cohort of HCWs regularly screened with PCR from April 2020 in the Friuli Venezia Giulia Region (NE, Italy) until the end of May 2021. Among HCWs, vaccination with BNT162b2 was associated with a sharp decline in the incidence of COVID-19 infection, with an IRR of 0.05 in vaccinate compared to non-vaccinated workers. We confirmed the 95% effectiveness of this vaccination for the prevention of SARS-CoV-2 infection.

## Data Availability

Data are available upon reasonable request.
